# How sample handling distorts telomere studies

**DOI:** 10.1038/s41598-025-08303-9

**Published:** 2025-07-01

**Authors:** Tijs K. Tournoy, Dries S. Martens, Julie De Backer, Paul Coucke

**Affiliations:** 1 Department of Cardiology, Ghent University Hospital, Corneel Heymanslaan 10, Ghent, 9000 Belgium; 2 Centre for Environmental Sciences, Hasselt University, Hasselt, 3500 Belgium; 3 Department of Cardiology and Center for Medical Genetics, Ghent University Hospital, Ghent, 9000 Belgium; 4 Center for Medical Genetics Ghent, Ghent University, Ghent, 9000 Belgium

**Keywords:** Genetics research, Telomeres, Genetics

## Abstract

Telomere length (TL) is investigated as a biomarker for aging and disease-susceptibility, but measurement using quantitative polymerase chain reaction (qPCR) faces challenges in accuracy and reproducibility. The potential impact of pre-analytical factors on TL measurements remains underexplored. We evaluated the impact of delayed blood processing, a typical feature in population studies. Blood samples from 35 adults were processed for buffy coat extraction either immediately or kept at 4 °C and processed after three and seven days (total *n* = 105). After processing, samples were stored at -80 °C. Relative TL was measured via qPCR and expressed as T/S ratio. Strikingly, delayed blood processing led to a significant increase in TL: the mean T/S ratio was 0.886 ± 0.205 at day 0, rising to 1.022 ± 0.240 at day 3 (*p* = 0.03) and to 1.190 ± 0.205 at day 7 (*p* < 0.001), corresponding to increases of 15% and 34%, respectively. Notably, TL correlated inversely with DNA integrity. These findings underscore the critical impact of delayed sample processing on TL measurements, emphasizing the need for consistent pre-analytical protocols to ensure accurate and reliable research outcomes. The impact of our findings is considerable as it may overshadow not only previously reported results but also real biological differences in TL between studied groups of patients.

## Introduction

Telomeres are the protective end caps of chromosomes composed of repetitive hexameric nucleotides (TTAGGG). Their primary function is to prevent the loss of DNA and chromosome fusion during cell replication. Telomeres shorten with each cell division until a critical length is reached, at which point cellular senescence is triggered^1–3^. The rate of telomere attrition is influenced by oxidative stress, which reflects exposure to various stressors. As telomeres shorten progressively, telomere length (TL) is studied as a biomarker for aging. Shorter telomeres are indeed associated with age related diseases such as coronary artery disease, cognitive impairment, type 2 diabetes mellitus, stroke and certain cancers^3^. In epidemiological research, TL is commonly measured in leukocytes which can serve as a proxy for many tissues^4,5^.

Various methods are available to quantify TL^6^, with quantitative polymerase chain reaction (qPCR) being the most commonly used technique. This method quantifies relative TL by comparing the amplification product of the telomere sequence (T) to that of a single-copy gene (S), resulting in the T/S ratio^6,7^. Several pre-analytical factors are known to influence TL measurements by qPCR. These include sample source, the number of freezing and thawing cycles, the DNA-extraction method and long-term storage conditions^8–10^. In addition, the accuracy of measurement can also be affected by the quality of DNA. This can be assessed by purity measurements evaluating the A260/230 and A260/280 absorbance ratios or by measuring the DNA Integrity Number (DIN)^8,9^.

In large population studies, blood collection procedures often do not allow immediate sample processing due to logistical reasons. In that case, blood samples may be stored at 4 °C for variable times before buffy coat isolation and DNA extraction. We hypothesized that the time between the drawing of blood and its processing could be an important and possibly underestimated factor. To the best of our knowledge, no concluding data is available on the impact of this common and controllable pre-analytical step. TL was found to be stable for four days when blood was stored at 4 °C or at room temperature. However this conclusion was based on the samples of a single patient^11^. To fill this gap, we assessed TL stability in a larger cohort of adults with predefined storage times of blood before separating and freezing the buffy coat at −80 °C. Blood was processed either immediately or stored at 4 °C for three or seven days before buffy coat isolation. The details of sample processing, DNA extraction and TL measurement are described thoroughly in the methods section.

## Results

**Fig. 1:**
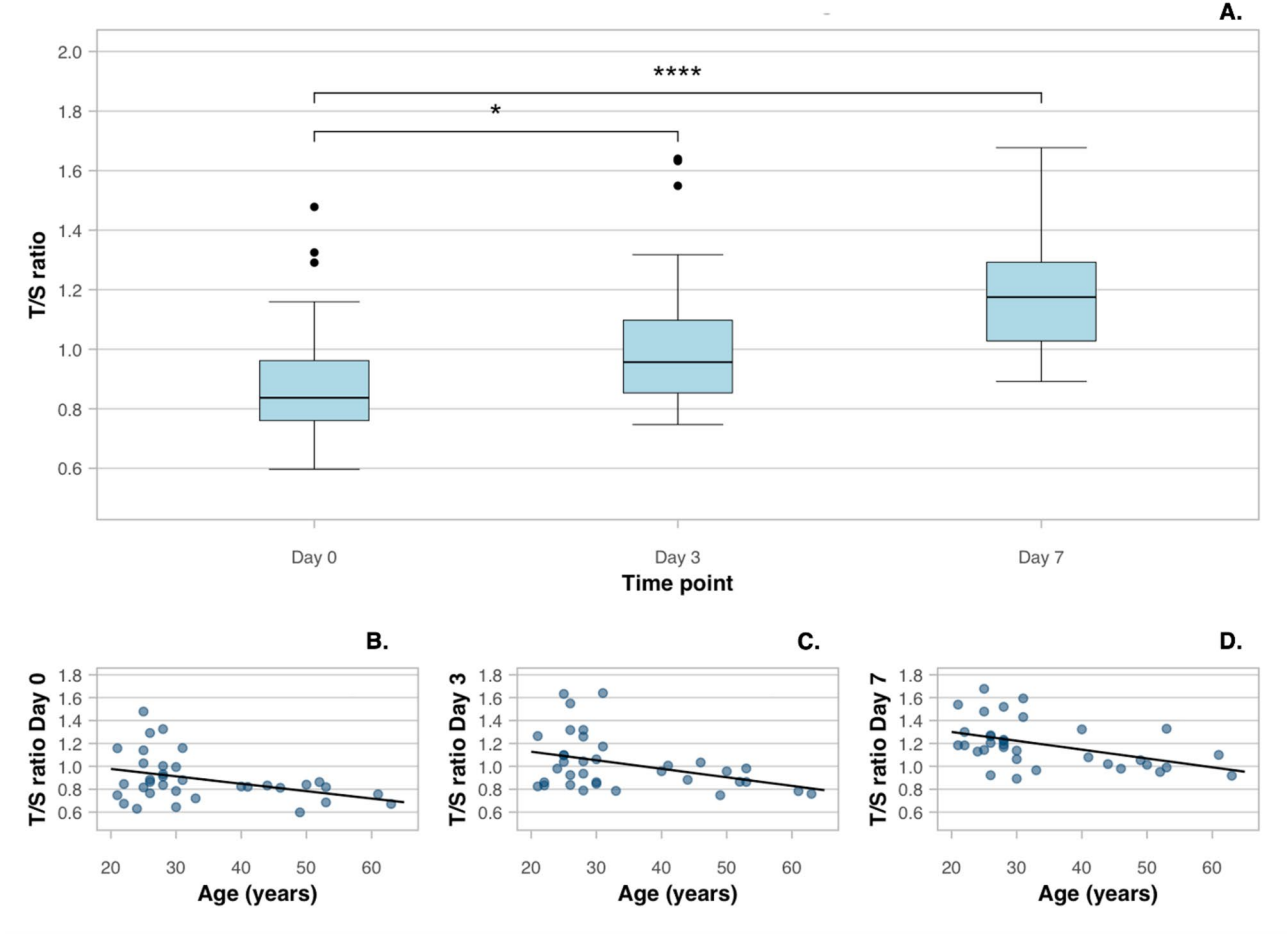
Evolution of telomere length. (**A)** Comparison of T/S ratio of samples processed at day 0, 3 and 7 (*: *p*<0.05, ****: *p*<0.0001). Boxes indicate interquartile range, horizontal lines in the boxes the median, and whiskers the range. (**B–D)** Correlation of T/S ratio and age at the different time points. (**B**) T/S ratio at day 0 (*r*^2^ = 0.146). (**C**) T/S ratio at day 3 (*r*^2^ = 0.141). (**D**) T/S ratio at day 7 (*r*^2^ = 0.210).

Blood samples were taken from 35 healthy adults (21 females, mean age 34 years, range 21–63 years). The mean relative TL expressed as a T/S ratio of immediately processed leukocytes was 0.886 ± 0.205 (range 0.597–1.478). At day 3 and day 7, this was 1.022 ± 0.240 (range 0.747–1.640) and 1.190 ± 0.205 (range 0.892–1.677) respectively (Fig. 1A). This corresponds with a significant increase in TL (ANOVA test *p* < 0.001) of 15% after three days and 34% after seven days. In addition to comparisons with baseline (day 0), a progressive increase in TL was observed from day 3 to day 7 (*p* = 0.006). As expected, TL was moderately inversely correlated to the chronological age of participants at day 0 (*r*^2^ = 0.146, *p* = 0.024), day 3 (*r*^2^ = 0.141, *p* = 0.026) and day 7 (*r*^2^ = 0.210, *p* = 0.006) (Fig. 1B–D).

**Fig. 2:**
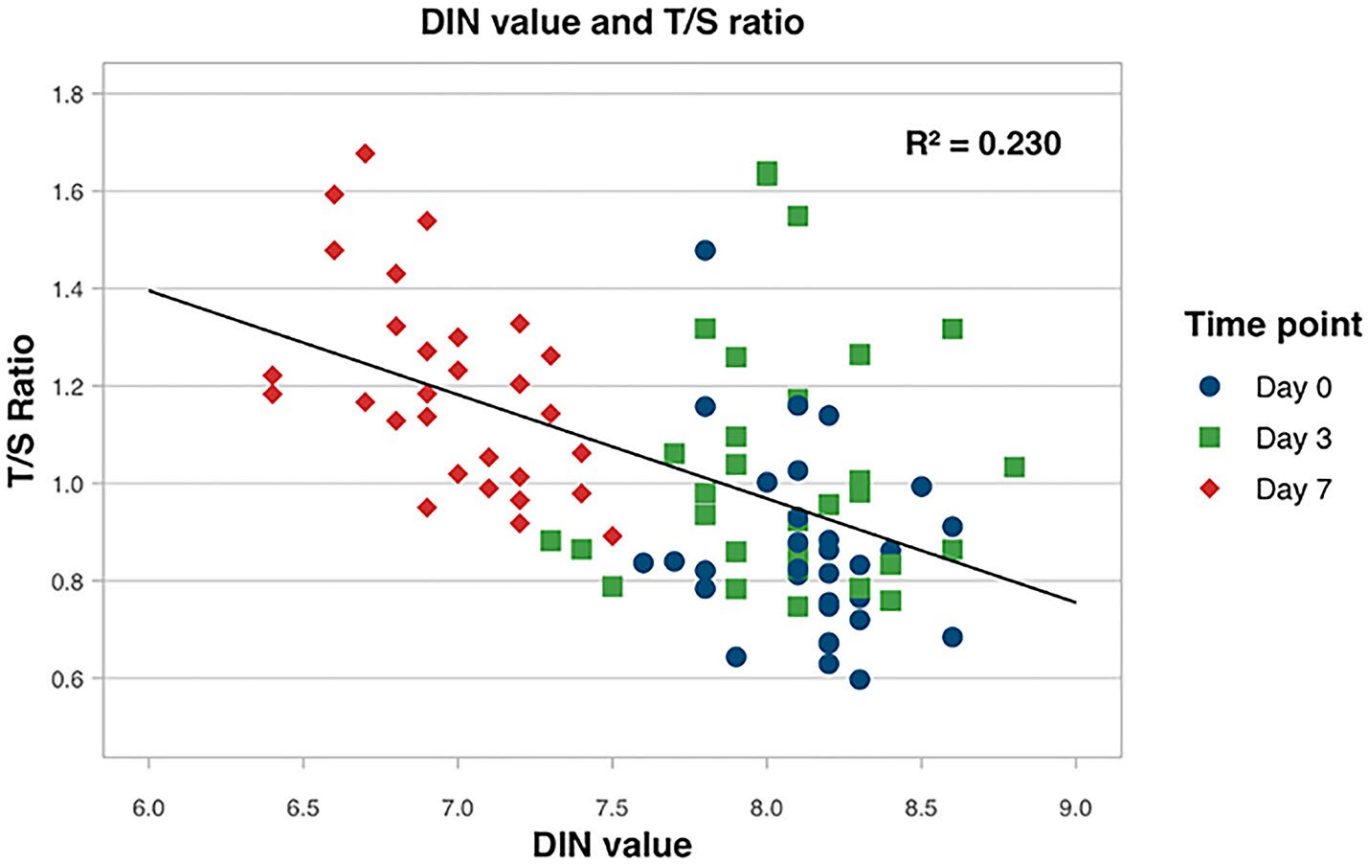
Telomere length and DNA integrity. Association between T/S ratio and DIN value, measured at the three time points. R^2^ = 0.230. DIN: DNA integrity number.

DNA-purity markers A260/230 and A260/280 ratio showed no statistically significant difference at the different time points (ANOVA test *p* = 0.526 for A260/280 ratio and *p* = 0.774 for A260/230 ratio). Integrity of DNA measured by DNA integrity number (DIN) was preserved at day 0 (DIN 8.14 ± 0.24) and day 3 (DIN 8.08 ± 0.34, *p* = 1.00). However, at day 7, DIN significantly dropped as compared to day 0 suggesting DNA degradation took place (DIN 6.99 ± 0.34, *p* < 0.002). A moderate inverse correlation between T/S ratio and DIN-value was observed (*r*^2^ = 0.230, *p* < 0.001, Fig. 2). Receiver Operating Characteristic analysis of a DIN cut-off of 7.5 to discriminate samples of day 0 and 3 from those processed at day 7 corresponded with an Area Under the Curve of 0.809 (sensitivity of 90% and specificity of 62%).

## Discussion

Our study demonstrates for the first time that delayed sample handling – an easily overlooked preanalytical factor – significantly affects relative TL measurements by qPCR. When blood is stored prolongedly at 4 °C before buffy coat extraction, the T/S ratio increases substantially to the extent that it may overshadow real biological differences. In recent years, the qPCR technique for TL measurement has been embraced and adapted by many for population studies. Variations in protocols have subsequently emerged using different qPCR systems, primers, reference genes, master mix compounds and qPCR cycles. However, the lack of universal consensus on the required quality controls complicates the comparability of results^8,12,13^. The measurement repeatability can be assessed by calculating the coefficient of variation (CV%). A lower CV% indicates greater reproducibility, with values below 10% generally considered acceptable within a laboratory. The CV% can also be used to compare qPCR results between labs. CV% between labs has been reported to be as high as 24%, reflecting differences in qPCR protocols and underscoring the need for caution when comparing results across studies^8,14^. The CV% and intraclass correlation coefficient (ICC) illustrate the reliability of measurement yet they are rarely reported. Details on sample processing or storage are seldomly documented^12^. Our data show that in addition to pre-analytical factors described before, inconsistencies in sample handling before long term storage will considerably distort TL results.

Different mechanisms may explain our observations. Telomeres can biologically elongate by the activation of the telomerase enzyme or by alternative lengthening of telomeres which is based on homologous recombination^15^. However, the activation of either mechanism during storage at 4 °C seems unlikely. Alternatively due to prolonged storage at 4 °C, telomeric DNA could become more euchromatic thus more accessible for PCR primers whereas the housekeeping gene, located in a non-terminal region of the genome, remains more protected. Indeed, samples stored for seven days exhibited a lower DIN thus the sample degradation might account for the elevated T/S ratio. In our data a DIN of 7.5 can be used to distinguish fresh samples (day 0 and day 3) from older samples (day 7). This cut-off could therefore be indicative for potentially less reliable TL results. Consistent with our findings, a recent study also reported that longer TL was associated with lower DNA integrity, demonstrating that lower DIN values can increase telomere length across a broad range of sample types^16^.

These new insights are particularly relevant as they could shed additional light on some of the unexpected and inconsistent findings in clinical studies^13,15^. For example, while shorter telomeres have been associated with cancer risk, the literature remains inconsistent^15,17^. A meta-analysis by Wentzensen et al. examined cancer risk by measuring tissue specific TL. Results of multiple studies were pooled adjusting for age, but without taking the method of telomere measurement into account or assessing the quality of TL measurement^17^. As illustrated above, such methodological variability necessitates caution when interpreting combined results. Similarly, substantial methodological heterogeneity in meta-analyses on the correlation between TL and smoking, hypertension or obesity was reported before^18–20^. The lack of standardization of the preanalytical factors, including sample handling, undoubtedly weakens the validity and comparability of findings across and within studies. Another challenge in using telomeres as a biomarker for aging is that the attrition of telomeres in longitudinal studies does not always seem to be linear. Namely, TL was remarkably reported to lengthen with advancing age in a subset of participants^21^. This observation has been attributed by the authors to a biological phenomenon but is at least partially due to inaccuracy of the TL measurement^21^. Moreover, in longitudinal studies, protocols might have evolved with unstandardized storage times and temperatures, DNA-extraction methods or qPCR protocols. These inconsistencies could further complicate the interpretation of the reported results, reinforcing the need for quality control in TL research^13,22^.

The aforementioned factors impact TL results measured by qPCR, yet they remain largely underreported in research articles^12,13^. We argue that the biological value of TL might therefore be underestimated due to technical variability and unreliable measurements. Indeed, qPCR can be highly precise and accurate when all (pre)analytical steps are carefully followed and described^23^. Our findings reinforce this statement and aid the dire need to optimize and standardize protocols^2,8^. We propose that in addition to consistent sample processing, assessing the DNA-integrity number could serve as a useful quality control measure to improve the reliability of TL measurements by qPCR. In conclusion, telomere measurement by qPCR holds great promise as an aging biomarker. It has also been a source of controversy since several preanalytical factors influencing the results have not received the necessary attention. Our study adds a new dimension, highlighting the important role of standardized sample storage times before processing. Given the magnitude of this effect, our findings help reframe some past unexpected or conflicting results and underscore the urgent need for stringent standardization in TL measurements.

## Materials and methods

### Sample collection and Preparation

The Ghent University Hospital Ethics Committee approved this study (reference number BC-5484 EC2019/0942), informed consent was obtained from all subjects and/or their legal guardian(s). All methods were carried out in accordance with relevant guidelines and regulations. Blood was drawn from 35 healthy adults, 21 females (60%) and 14 males. Blood was stored in three separate 4.0 mL tubes containing ethylenediaminetetraacetic acid (EDTA) per adult. Each tube was processed at a different time point, for one buffy coat was generated immediately (within two hours) after drawing the blood, where the other two whole blood samples were stored for three (72 h ± 2 h) and seven days (168 h ± 2 h) at 4 °C. At the time of processing, samples were centrifuged using the Eppendorf 5810 centrifuge for 10 min at 3000 rpm, corresponding with a relative centrifugal force of 1740 g. Centrifugation was performed at room temperature. The rotor used was a swinging-bucket type, and brake settings were applied to stop the rotor immediately after centrifugation. One buffy coat was isolated, aliquoted in two cryotubes and stored at −80 °C until DNA-extraction was performed on all samples 2 weeks later. Therefore, the only difference between samples was the time before the buffy coat was separated and stored at −80 °C. DNA was extracted using QIAmp DNA Blood Mini Kit (Qiagen) and afterwards stored at −20 °C until further analysis (8 weeks). DNA concentration and purity (A260/230 and A260/280 ratio) were measured using the Lunatic spectrophotometer (Unchained Labs, CA, USA), following the manufacturer’s protocol. DNA integrity number was measured on all samples using the Agilent TapeStation (Agilent Technologies) according to the manufacturer’s instructions.

### Telomere length measurement

Average relative telomere length was measured using a modified singleplex qPCR adapted from Cawthon, 2002 and 2009^7,24^. To ensure a uniform DNA input of 5 ng for each qPCR reaction, samples were diluted and checked using the Qubit™ dsDNA High Sensitivity Assay Kit (Life Technologies, Europe) using the Qubit™ Flex Fluorometer (Life Technologies, Europe). All samples were measured in triplicates on a QuantStudio 5 real-time PCR system (Applied Biosystems) in a 384-well format. First, a single copy gene (human β globin) reaction was performed containing 5 ng DNA template, 1x KAPA SYBR^®^ FAST, Low ROX™ master mix (Kapa Biosystems, Merck), 450 nM HBG1 primer (GCTTCTGACACAACTGTGTTCACTAGC), and 450 nM HBG2 primer (CACCAACTTCATCCACGTTCACC). Cycling conditions were as follows: 1 cycle at 95 °C for 3 min, 40 cycles at 95 °C for 3 s, and 58 °C for 15 s. Second, a telomere-specific reaction was performed, containing 5 ng DNA template, 1x KAPA SYBR^®^ FAST, Low ROX™ master mix (Kapa Biosystems, Merck), 2mM DTT, 100 nM TelG primer (ACACTAAGGTTTGGGTTTGGGTTTGGGTTTGGGTTAGTGT), and 100 nM TelC primer (TGTTAGGTATCCCTATCCCTATCCCTATCCCTATCCCTAACA). Cycling conditions were as follows: 1 cycle at 95 °C for 3 min, 2 cycles at 94 °C for 3 s and 49 °C for 15 s, and 30 cycles at 94 °C for 3 s, 62 °C for 5 s, and 74 °C for 10 s. For each PCR reaction (telomere and single-copy gene), all samples were run on a single 384-well plate to avoid inter-plate variation. On each run, PCR efficiency was evaluated using a standard 6-point serial diluted DNA standard curve (efficiency was 101% for Tel, and 98% for HBG with an R^2^ > 0.99). The final average relative TL was calculated as a normalized relative quantity (NRQ) using the qBase software (Biogazelle, Zwijnaarde, Belgium). First, the relative quantity (RQ) was calculated based on the delta-Cq method for telomere (T) and single-copy gene (S) obtained Cq values. As the choice of a calibrator sample (sample to which subsequent normalization is performed, delta-delta-Cq) strongly influences the error on the final RQs (as a result of the measurement error on the calibrator sample), normalization was performed to the arithmetic mean quantification values for all analyzed samples, which results in the NRQ. Mathematical calculation formulas to obtain RQ, and NRQ are provided by Hellemans et al.^25^. The method precision is shown by an intra-assay ICC of 0.961 (0.944–0.973) and a CV% of 4.11%, confirming high consistency and low variability of measurement. Additionally, to further support reproducibility, the ICC and CV were calculated separately for the T and S reactions. The T reaction showed an ICC of 0.948 (0.925–0.963) and a CV% of 0.47%, while the S reaction demonstrated an ICC of 0.897 (0.852–0.929) and a CV% of 0.26%.

### Statistics

SPSS software (IBM, Version 29.0. Armonk, NY, US) was used for statistical analysis. ANOVA test was applied to compare T/S ratios between groups, with a Bonferroni correction for multiple comparisons. Correlations were estimated by the Spearman’s rank order correlation coefficient. Graphs were generated using RStudio (version 4.3.3, R Core Team).

## Data Availability

The datasets generated during and/or analysed during the current study are available from the corresponding author on reasonable request.
